# Genetic Polymorphisms in VEGFR Coding Genes (*FLT1*/*KDR*) on Ranibizumab Response in High Myopia and Choroidal Neovascularization Patients

**DOI:** 10.3390/pharmaceutics14081555

**Published:** 2022-07-26

**Authors:** David Blánquez-Martínez, Xando Díaz-Villamarín, Sonia García-Rodríguez, Alba Antúnez-Rodríguez, Ana Pozo-Agundo, Luis Javier Martínez-González, José Ignacio Muñoz-Ávila, Cristina Lucía Dávila-Fajardo

**Affiliations:** 1 Pharmacy Department, Hospital Universitario de Ceuta, 51003 Ceuta, Spain; david.blanquez.sspa@juntadeandalucia.es; 2 Pharmacology Department, University of Granada (UGR), 18016 Granada, Spain; 3 Instituto de Investigación Biosanitaria de Granada (ibs.Granada), 18016 Granada, Spain; garciarodriguez.sonia@gmail.com (S.G.-R.); alba.antunez@genyo.es (A.A.-R.); ana.pozo@genyo.es (A.P.-A.); 4 Genomics Unit, Centre for Genomics and Oncological Research: Pfizer, University of Granada, Andalusian Regional Government (GENYO), 18016 Granada, Spain; luisjavier.martinez@genyo.es; 5 Ophthalmology Department, Instituto de Investigación Biosanitaria de Granada (ibs.Granada), Hospital Universitario Clínico San Cecilio, 18016 Granada, Spain; josei.munoz.sspa@juntadeandalucia.es; 6 Pharmacy Department, Instituto de Investigación Biosanitaria de Granada (ibs.Granada), Hospital Universitario Virgen de las Nieves, 18016 Granada, Spain; cristinal.davila.sspa@juntadeandalucia.es

**Keywords:** pharmacogenetics, myopia, ranibizumab, precision medicine, VEGFR, anti-VEGF, *FLT1*, *KDR*

## Abstract

A severe form of myopia defined as pathologic/high myopia is the main cause of visual impairment and one of the most frequent causes of blindness worldwide. It is characterized by at least 6 diopters or axial length (AL) of eyeball > 26 mm and choroidal neovascularization (CNV) in 5 to 10% of cases. Ranibizumab is a humanized recombinant monoclonal antibody fragment targeted against human vascular endothelial growth factor A (VEGF-A) used in the treatment of CNV. It acts by preventing VEGF-A from interacting with its receptors (VEGFR-1 and -2) encoded by the *FLT1* and *KDR* genes. Several studies found that the *KDR* and *FLT1* genotypes may represent predictive determinants of efficacy in ranibizumab-treated neovascular age-related macular degeneration (nAMD) patients. We performed a retrospective study to evaluate the association of single nucleotide polymorphisms (SNPs) in VEGFR coding genes with the response rate to ranibizumab in patients with high myopia and CNV. In the association study of genotypes in *FLT1* with the response to ranibizumab, we found a significant association between two *FLT1* variants (rs9582036, rs7993418) with ranibizumab efficacy at the 12-month follow-up. About the *KDR* gene, we found that two *KDR* variants (rs2305948, rs2071559) are associated with best-corrected visual acuity (BCVA) improvement and *KDR* (rs2239702) is associated with lower rates of BCVA worsening considering a 12-month follow-up period.

## 1. Introduction

Myopia is an eye disease with a varied geographic prevalence. In Asian countries, its prevalence is up to 70–90%, whereas in western Europe, it is 25–50% [1,2,3]. The most severe form of myopia, defined as pathological/high myopia, is the main cause of visual impairment and one of the most frequent causes of blindness worldwide [4]. Patients with pathological myopia are characterized by an axial length ≥26 mm or a refractive error of −6.0 diopters or more [1,5,6]. These patients have progressive and excessive globe elongation with retinal and choroidal thinning, peripheral retinal degeneration, and an increased risk of retinal detachment, cataracts, glaucoma, and myopic choroidal neovascularization (mCNV) [7,8].

mCNV develops in 5–10% of pathological myopia patients and it is one of the most vision-threatening complications of these patients [1,9,10]. Furthermore, pathologic/high myopia is the most common cause of CNV in individuals aged 50 years or younger and the second cause of CNV after nAMD [11].

mCNV is characterized clinically by retinal hemorrhage with or without exudation. This hemorrhage, after its resolution, may leave an area of chorioretinal atrophy or a grayish-white pigmented scar (Fuchs spot). mCNV is different in young or aged patients. Young patients usually show small classic lesions located close to the fovea. These lesions can cause rapid visual loss with or without metamorphopsia and/or central scotoma. Moreover, in aged individuals, mCNV is more extensive and exudative; it may lead to chorioretinal scar formation similar to nAMD. Some cases of mCNV may regress spontaneously with minimal impact on vision; however, without appropriate treatment, most eyes will have a poor visual outcome [11].

The most common treatment for mCNV includes thermal laser photocoagulation, verteporfin photodynamic therapy (vPDT), and surgery, specifically removing the mCNV [12,13,14]. Laser photocoagulation treatment can result in permanent retinal damage, causing scotoma increase, scarring, and visual loss over time. Moreover, recurrent CNV may arise at the margins of previously laser-treated areas. This may be due to rupture of the retinal pigment epithelium and Bruch’s membrane stimulating new CNV lesions in some patients [15,16].

In 2001, the results of the VIP study based on the use of vPDT in mCNV were published, which showed that it was capable of maintaining visual acuity (VA) at 12 months [17], although no significant difference was observed in the primary outcome at 24 months [18]. Furthermore, the development of long-term chorioretinal atrophy based on the use of vPDT in mCNV was observed in another study [19]. The results of the surgery were not good either [14].

The VEGF is a potent proangiogenic factor that stimulates CNV development. Following binding to VEGFR, endothelial cells are stimulated to proliferate, migrate, and express matrix-degrading proteases, causing vascular instability, leakage, and finally angiogenesis [11].

The introduction of monoclonal antibodies directed against VEGF in the treatment of mCNV was a real breakthrough. Ranibizumab (Lucentis^®^, Novartis Pharma AG; Stein, Switzerland) is a humanized recombinant monoclonal antibody fragment targeted against human VEGF-A. It binds with high affinity to VEGF-A, thereby preventing binding of VEGF-A to VEGFR-1 and -2 at the endothelial cell surface, thus inhibiting cell division, migration, and angiogenesis; increasing apoptosis; and reducing the permeability/leakage of the vasculature [20,21].

The RADIANCE, a phase III, 12-month study that evaluated Ranibizumab and PDT (verteporfin) in mCNV, proved that ranibizumab has good efficacy and safety, with a significant and maintained gain in VA. Furthermore, a relatively low number of injections are required to treat most patients with mCNV. Over 60% of patients did not require any injections from month 6 onwards, and more than 50% required only 1 or 2 injections over 12 months. Although, 34.5% required 3–5 injections, and 14.7% needed 6–12 injections during the 12-month study period. This suggests a need to understand factors such as genetic influence that may predict treatment response and the development of individualized treatment regimens [22].

**Figure 1:**
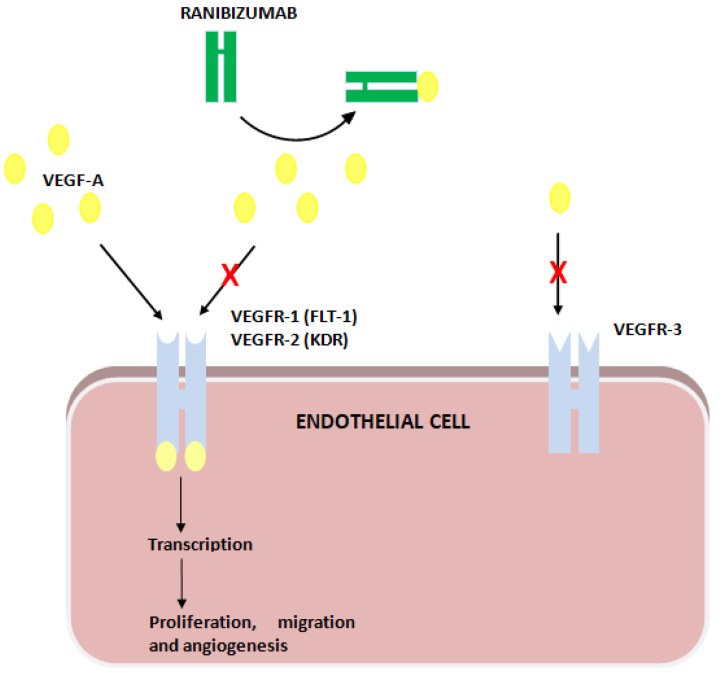
Ranibizumab’s action on VEGF receptors (VEGFR).

Ranibizumab binds VEGF-A, impeding its action on VEGFR-1 and -2. VEGFR-1 and -2 are encoded by *FLT1* and *KDR*, respectively (Figure 1).

Cobos et al. [23] found that *FLT1* (VEGFR-1 coding gene) rs7993418 is associated with the ranibizumab treatment response in nAMD patients. Furthermore, Beuselinck B. et al. [24,25] and Dornbusch J. et al. [26] found that for sunitinib, used in the treatment of neoplastic pathologies such as metastatic kidney cancer by inhibiting neoangiogenic processes, *FLT1* (rs9582036) is a predictive biomarker of treatment response.

The VEGFR-2 receptor, encoded by the *KDR* gene, is a high-affinity receptor tyrosine-kinase responsible for most of the angiogenic- and permeability-enhancing effects of VEGF-A. Thus, *KDR* (VEGFR-2 coding gene) variants are candidates for a possible genetic influence susceptibility to anti-VEGF therapy [27]. Even Lazzeri et al. [27] found that the *KDR* (VEGFR-2) rs2071559 genotype may be a predictive determinant of short- and long-term functional and anatomical outcomes in ranibizumab-treated nAMD patients. Hermann et al. [28] concluded that genetic polymorphisms in the *KDR* gene significantly influence the visual outcome in patients receiving ranibizumab treatment for nAMD.

Among our patients, when diagnosed with mCNV and treated with ranibizumab, *ARMS2* (rs10490924) and *CFH* (rs1061170) SNPs were associated with response [29]. The *ARMS2* (rs10490924) G allele and GG genotype led to a better response to ranibizumab at the 6-month follow-up. In contrast, the *CFH* (rs1061170) T allele and TT genotype were associated with higher rates of BCVA worsening at the 1-month follow-up.

In summary, ranibizumab is used in the treatment of high myopia and CNV patients. It binds VEGF-A, impeding its binding to VEGFR-1 and -2, encoded the by *FLT1* and *KDR* genes. Thus, the genetic polymorphisms in both genes previously associated with interindividual differences in drug responses may also affect the ranibizumab response in mCNV.

This study aimed to evaluate the association of SNPs in the VEGFR (VEGFR-1 (*FLT-1*) and VEGFR-2 (*KDR*)) coding genes with the response rate to ranibizumab in patients with mCNV.

## 2. Materials and Methods

In this study, we assessed the same cohort of patients as the previous study by Blánquez-Martínez et al. [29] but with a 12-month follow-up period.

In summary, we carried out a retrospective study, recruiting patients between 2014 and 2019 with high myopia and CNV treated with ranibizumab at our hospital. Among these patients, we assessed the association of genetic polymorphisms in *KDR* and *FLT1* with the response (BCVA improvement and worsening) to ranibizumab. We also recruited a control group with high myopia but no CNV to take care of the association of the included SNPs with the disease instead of the response to ranibizumab.

Specific characteristics about the inclusion/exclusion criteria, considered criteria for high myopia diagnosis, patient management, data management including definitions of BCVA “improvement” and “worsening”, and DNA extraction and genotyping have been previously published in the study by Blánquez-Martínez et al. [29].

This study was conducted in accordance with the Declaration of Helsinki and approved by the Ethics Committee of Granada (Spain) “*CEIM/CEI Provincial de Granada*” (approval code: 0085-N14; 26 May 2014).

### 2.1. Procedures for the Inclusion of Genetic Variants in the Study

We considered for inclusion those genetic variants in genes encoding the VEGFR-1 and -2 (*KDR* and *FLT1*) that had previously been proved to affect drug efficacy or toxicity. To do this, we searched PharmGKB for information about both genes. We considered for inclusion those genetic variants with a related annotation reporting the association with drug responses (efficacy or toxicity) in at least one publication and excluded those genetic variants with a minor allele frequency (MAF) lower than 10%. This means that we considered for inclusion the *KDR* rs2071559, rs2239702, rs1870377, rs34231037, rs2305948, and rs7667298 variants; and *FLT1* rs664393, rs7993418, rs9554320, and rs9582036. Finally, we excluded *KDR* rs34231037 since it was the only one with a known MAF lower than 10% in the Iberian Peninsula population.

### 2.2. DNA Extraction and Genotyping

For genotyping, we took 4 saliva samples with sterile cotton swabs from each recruited patient. We isolated the DNA using standard procedures and DNA extraction was carried out following the method by Freeman et al. [30] with modifications by Gomez-Martín A. et al. [31]. We genotyped SNPs using KASP assay technology (LGC Genomics, Hoddesdon, Hertfordshire, UK) following the manufacturer’s instructions and these were analyzed with the KlusterCaller Software (LGC Genomics, Hoddesdon, Hertfordshire, UK). The call rate for all tested SNPs was >98%. Quality control for the genotyping results was achieved with negative controls and randomly selected samples included as duplicates.

### 2.3. Statistical Analysis

First, we carried out a descriptive analysis of the clinical parameters (Table 1) and calculated the distribution (number of patients and percentage) of genotypes and the minor allele frequency (MAF) of the included SNPs for both the control and study groups (Table 2). Then, we studied the Hardy–Weinberg (H-W) equilibrium of each SNP by group and for the total number of patients, and we performed an LD analysis of the genetic variants in each gene. Finally, the distribution of genotypes between the control and study groups was compared to assess the association of each SNP with CNV.

The main aim was to study the association of included SNPs and BCVA improvement or worsening at the 12-month follow-up. In this regard, we carried out both allele and genotype comparison analyses. In the genotype analysis, we used each genetic model (recessive, dominant, co-dominant, and log-additive). We also performed a haplotype association study.

We used the Chi-square test or Fischer exact test. We calculated the odds ratio (OR) and *p*-values. *p*-values < 0.05 were considered statistically significant. The Bayesian information criterion (BIC) and Akaike information criterion (AIC) were calculated for each genetic model and SNP–response association study.

The descriptive analysis (Table 1), MAFs, genotypic distribution, and its comparison among groups (Table 2) were performed using R commander. The association studies and H–W equilibrium analysis were conducted using the SNPstats online tool [32].

The sample size calculation was based on that shown in the previous research article evaluating the influence of *VEGFR* variants on ranibizumab response in non-high myopia patients [23,24,25,26,27,28]. Furthermore, we recruited the total number of patients with high myopia and treated with ranibizumab in our hospital for five years. Both the sample size and statistical influence of SNPs on genetic diseases were studied in several works that investigated the impact on pathological phenotypes [33,34,35,36].

## 3. Results

In this study, we assessed the same cohort of patients published by Blánquez-Martínez et al. [29], including new genetic variants and extending the follow-up period to 12 months.

As commented in this previous study [29], we recruited *n* = 100 patients and *n* = 113 eyes diagnosed with high myopia and CNV that were treated with ranibizumab. We did not find an adequate DNA concentration for genotyping in one patient (*n* = 1 eye). Finally, *n* = 99 patients and *n* = 112 eyes were included in the study. Among the patients in the study group, 75% were women, with a mean age of 57.5 ± 13.9 years. Most of the studied eyes showed a juxta foveal CNV location (66.1%) and were not previously treated with LP/PDT (92%). On the other hand, *n* = 30 (26.8%) and *n* = 8 (7.1%) eyes presented a sub-foveal and extra-foveal location, respectively, and *n* = 8 (7.1%) had been treated with LP and *n* = 1 (0.9%) with PDT. The mean BCVA (logMAR) at baseline was 0.62 ± 0.48 and 0.34 ± 0.38 at the 12-month follow-up.

Regarding the control group, we recruited *n* = 116 patients and *n* = 219 eyes. In this group, *n* = 7 eyes could not be assessed. Among them, in 4 patients (*n* = 4 eyes), we could not obtain an adequate DNA concentration to investigate the needed genotypes; in *n* = 2 patients (*n* = 2 eyes), saliva samples were lost; in and 1 patient (*n* = 1 eye), mCNV was diagnosed one month after being recruited. This patient was transferred from the control to the treatment group.

The baseline characteristics of the recruited patients (study and control group) and the follow-up characteristics of the study group are shown in Table 1.

### 3.1. Genotypic Distribution, H–W Equilibrium, Association of Genetic Variants with High Myopia and CNV, and Linkage Disequilibrium Analysis

Among the included genetic variants, *FLT1* C > T (rs664393) was the only one that showed a minor allele frequency (MAF) lower than 0.1, with no patients carrying the recessive homozygous genotype in the study group. None of the studied SNPs showed significant differences in the H–W equilibrium analysis, and the *FLT1* C > A (rs9554320) was the only one that showed differences in the comparison of the genotypic distribution between the control and study groups (Table 2).

In the linkage disequilibrium (LD) analysis, regarding the *FLT1* variants, we found that rs664393 was not linked to any other variant; however, three other included variants were linked among them (D’ > 0.97; *p* < 0.001) (Figure S1). Among the *KDR* variants, rs2305948 was not linked to any other variant and the other four variants were linked among them (*p* < 0.01) (Figure S2).

### 3.2. Association of Genetic Polymorphisms with Response to Ranibizumab

#### 3.2.1. Genotype Association Study with Response

In the association study of the genotypes in *FLT1* with the response to ranibizumab (Table 3), we found that the *FLT1* (rs9582036) CC genotype was associated with lower rates of BCVA improvement at the 12-month follow-up (recessive model: CC vs. CA or AA; OR = 0.2; 95%CI = 0.04–0.9; *p* = 0.032). On the other hand, we did not find a significant association between the *FLT1* (rs9582036) CC genotype with higher rates of worsening. Additionally, in *FLT1*, we found that the *FLT1* (rs7993418) AA genotype was associated with higher rates of worsening (dominant model: AA vs. AG or GG; *p* = 0.013). Finally, we found no significant association of *FLT1* (rs9554320) with BCVA improvement or worsening at the 12-month follow-up. We do not provide results about *FLT1* (rs664393) since we did not identify patients who were carrying the recessive homozygous genotype.

About the *KDR* gene (Table 4), *KDR* (rs2305948) showed a significant association with BCVA improvement in the log-additive model (*p* = 0.049). Based on the other genetic models, it seems that the CC genotype is related to higher rates of BCVA improvement (dominant model: CC vs. CT or TT; OR = 0.35; 95%CI = 0.11–1.11; *p* = 0.055). Additionally, *KDR* (rs2071559) was associated with BCVA improvement among ranibizumab-treated patients (over-dominant model: OR = 2.91; 95%CI= 1.19–7.08; *p* = 0.015). Regarding *KDR,* among our patients, the *KDR* (rs2239702) GG genotype was associated with lower rates of BCVA worsening (dominant model: GG vs. GA or AA; OR= 0.15; 95%CI= 0.02–1.31); *p* = 0.044).

Table 3 and Table 4 show the detailed results of the association study with the response (BCVA improvement or worsening) of all SNPs in *KDR* and *FLT1* included in this study.

#### 3.2.2. Alleles Association Study with Response

In the allele association study with the response, we found that the *KDR* (rs2239702) A allele was associated with higher rates of BCVA worsening (OR = 3.33; 95%CI = 1.03–10.78; *p* = 0.035) (Table 5). In contrast, it was the only SNP found to be related to ranibizumab response (BCVA improvement or worsening) among our patients. On the other hand, the *KDR* (rs2305948) T allele and (rs1870377) A allele showed close-to-significant results (*p* = 0.073 and *p* = 0.097, respectively) for their association with BCVA improvement and ranibizumab treatment.

### 3.3. Haplotype Association Study with Response

In the haplotype association analysis with response to ranibizumab, we found that no haplotypes for *FLT1* were associated with BCVA improvement nor with worsening (Table S1). For *KDR*, and according to the allele association study with the response, we found the most common haplotype (rs2239702 G; rs2305948: C; rs7667298: C; rs1870377: T and rs2071559: T) was associated with lower rates of worsening (OR= 0.09; 95% CI= 0.01–0.86; *p* = 0.039) compared to the ACTTC haplotype, carried by 21.9% of the patients (Table S2).

## 4. Discussion

Ranibizumab is an anti-VEGF drug used in the treatment of mCNV among other pathologies. Many genetic polymorphisms have been associated with the response to this drug. Among AMD patients, *CFH* (rs1061170) and *ARMS2* rs10490924 were related to interindividual differences in the response to anti-VEGF drugs [37,38]. Additionally, among AMD patients, genetic polymorphisms in *VEGFA* (rs3025000, 833069, and rs699947) [37,39,40,41], *NRP1* (rs2070296) [42], and *CXCL8* (rs4073) [27] are associated with interindividual differences in the response to ranibizumab. Polypoidal choroidal neovascularization is usually considered a sub-type of CNV. In patients with CNV and/or PCV, *VEGFA* (rs2010963) [43], *HTRA1* (rs11200638) [44], and especially *CFH* (rs1061170) and *ARMS2* (rs10490924) [45] were also found to be associated with ranibizumab efficacy.

The influence of genetic polymorphisms on the ranibizumab response among mCNV patients has not been widely studied. Among our patients, we found in a previous study [29] that *CFH* (rs1061170) and *ARMS2* (rs10490924) are related to ranibizumab efficacy at the 1- and 6-month follow-up. The *CFH* (rs1061170) C allele was associated with lower BCVA worsening and BCVA improvement, the TT genotype was associated with BCVA worsening, and the CC genotype was associated with BCVA improvement. About *ARMS2* (rs10490924), the G allele and GG genotype showed an association with BCVA improvement.

On the other hand, in this previous study, we still found patients that did not meet the expected progress of the illness based on our results. Thus, we considered a study of the genetic polymorphism in *KDR* and *FLT1* considering a larger follow-up time (12 months). SNPs in these genes have not been previously studied in mCNV patients treated with ranibizumab.

This study had some limitations. It is a retrospective observational study; this means that we did not assess the clinical impact of the studied genetic polymorphisms on the ranibizumab response in daily clinical conditions. However, this would not be ethical since these genetic polymorphisms have not been associated with differences in the ranibizumab response. We recruited a control group without collecting data about BCVA improvement/worsening because this was only to control the possible association of genetic polymorphisms with mCNV, not with ranibizumab efficacy. We recruited n= 100 patients; the study cohort is low, and it should be increased in further studies. On the other hand, this was the total number of patients with mCNV treated with ranibizumab in our hospital in a 5-year period. In the same regard, we did not perform a multivariant analysis considering the combined genetic and clinical parameters. We did not perform a multiple test analysis since it would increase the risk of type I errors and the low number of recruited patients did not allow a Bonferroni correction to be performed. Regarding the assessment of eyes/patients, we included both eyes of patients with bilateral treatment considering that they might progress in a different way depending on the expression/silencing of PGx variants.

Our results still support the need for further studies, considering a clinical trial including combined PGx, clinical parameters (e.g., previous treatments such as LP and PDT), in a multivariant analysis, and in a larger cohort that includes patients from different populations.

### 4.1. FLT1 Genetic Polymorphisms and Ranibizumab

*FLT1* (fms-related receptor tyrosine kinase 1) is a protein-coding gene encoding a member of the VEGFR family that binds to VEGFR-A and VEGFR-B with an important role in angiogenesis and vasculogenesis. This receptor is expressed in vascular endothelial and placental trophoblast cells and peripheral blood monocytes. Ranibizumab binds VEGFA; thus, genetic variants in *FLT1* may result in conformational changes in the receptor or differences in VEGFR1 administration and expression. *FLT1* (rs9582036) is an intron variant and *FLT1* (rs7993418) is a synonymous variant. Thus, in both cases, they may affect the administration or expression of VEGFR1, affecting how VEGFA binds this receptor and, in some way, how ranibizumab impedes the effect of VEGFA on VEGFR1.

Many genetic variants in *FLT1* are associated with interindividual differences in the response to different treatments, especially in oncological patients [24,25]; however, there is only one study that has reported results about *FLT1* SNPs’ influence on ranibizumab efficacy [46]. In this study, Lotery et al. assessed the association of *FLT1* (rs12877323), among more than 450 SNPs in different genes with changes in the total retinal thickness (TRT) at the 3-, 6-, 9-, and 12-month follow-up in AMD patients without finding significant results. Among our patients, we did not study this SNP since this had not been previously associated with any drug response.

As commented above in the results section, among the included SNPs, we found a significant association between two *FLT1* variants (rs9582036, rs7993418) with ranibizumab efficacy in mCNV at the 12-month follow-up in the genotype association study. Even *FLT1* (rs7993418) showed a significant association with ranibizumab response in the allele association study (*p* = 0). In this regard, we did not find patients carrying the *FLT1* (rs7993418) G allele and BCVA worsening.

As we can see (Table 2), none of these two SNPs showed differences between the study and control group and there is a theoretical background supporting the correlation between this gene and ranibizumab efficacy, thus *FLT1* (rs9582036, rs7993418) may be considered a genetic marker of ranibizumab efficacy in mCNV patients.

### 4.2. KDR Genetic Polymorphisms and Ranibizumab

*KDR* (kinase insert domain receptor) is another protein-coding gene encoding a member of the VEGFR family. This receptor works as the main mediator of VEGF-induced endothelial proliferation, survival, migration, tubular morphogenesis, and sprouting. The *KDR* (rs2305948) is a missense variant and *KDR* (rs2239702) is an upstream gene variant. As ranibizumab binds VEGFA, these variants may result in conformational changes or differences in VEGFR1 administration and expression, respectively, leading to interindividual differences in the response to ranibizumab.

*KDR* variants, similar to *FLT1* variants, were also associated with interindividual differences in the response to oncological treatments [47,48,49,50,51] and also to clopidogrel response [52]. About ranibizumab, Lazzeri et al. [27] concluded that the *KDR* (rs2071559) CC genotype revealed a better functional response as measured by the mean retinal sensitivity (*p* = 0.034) in AMD patients. On the other hand, Smailhodzic et al. [37] did not find significant results about the association of *KDR* (rs2071559, rs7671745) SNPs with ranibizumab response at a 3-month follow-up, but the discrepancies with our results may be explained by the follow-up period (3 vs. 12 months).

Among our patients, considering the 12-month follow-up period, we found that the *KDR* (rs2305948) CC genotype was associated with BCVA improvement (log-additive model: *p*-value= 0.049; dominant model: CC vs. CT or TT, OR= 0.35; 95%CI= 0.11–1.11; *p* = 0.055). Additionally, the *KDR* (rs2239702) GG genotype was associated with lower rates of BCVA worsening (dominant model: GG vs. GA or AA; OR= 0.15; 95%CI= 0.02–1.31; *p* = 0.044).

Furthermore, in the allele association study with the response, we found some results supporting this. The rs2239702 A allele was associated with higher rates of BCVA worsening (*p* = 0.035), the rs2305948 T allele was almost associated with BCVA improvement (*p* = 0.073), and the haplotype combining the rs2239702 A allele and rs2305948 C allele was related to higher rates of worsening (*p* = 0.039).

The rs2305948 was the only variant not linked to any of the others included and it was related to ranibizumab efficacy. Among the other SNPs in *KDR* included in the analysis, they were linked among them and we observed different associations with ranibizumab efficacy. Additionally, in the haplotypes study, we found that the most common haplotype (rs2239702 G; rs2305948: C; rs7667298: C; rs1870377: T and rs2071559: T) was associated with lower rates of BCVA worsening (OR = 0.09; 95% CI = 0.01–0.86; *p* = 0.039) compared to the ACTTC haplotype, carried by 21.9% of patients. Because of this, we might consider the ACTTC haplotype as a predictor of a worse response to ranibizumab, not a single analysis of SNPs.

## 5. Conclusions

Based on our results, *FLT1* (rs9582036, rs7993418) variants may be genetic markers of ranibizumab efficacy in mCNV patients, and *KDR* (rs2305948, rs2239702) SNPs may be single predictors of ranibizumab efficacy in mCNV patients; however, the analysis of the ACTTC haplotype should be considered.

## Figures and Tables

**Table 1:** Baseline and follow-up descriptive analysis.

Variable	Ranibizumab Mean ± SD or *n* (%)	
	Study	Control
**Baseline characteristics**		
Total eyes (*n*)	112	219
Mean age (years)	57.5 ± 13.9	57.5 ± 15.1
Sex (Male:Female; %)	25:75	32:68
Mean SERE (Diopters)	12.1 ± 5.4	12.3 ± 4.9
Mean AL (mm)	28.8 ± 2.1	28.3 ± 1.9
Affected eye		
RE	61 (54.5)	
LE	51 (45.5)	
CNV Location		
Subfoveal	30 (26.8)	
Juxtafoveal	74 (66.1)	
Extrafoveal	8 (7.1)	
Previous treatment		
None	103 (92)	
LP	8 (7.1)	
PDT	1 (0.9)	
BCVA (logMAR) at BL	0.62 ± 0.48	
**12-month follow-up characteristics**		
BCVA (logMAR)	0.34 ± 0.38	
BCVA change (logMAR)	−0.28 ± 0.37	
BCVA improvement:		
Improvement	81 (72.3)	
Non improvement	24 (21.4)	
Worsening	7 (6.3)	

SERE = spherical equivalent refractive error; BCVA = best-corrected visual acuity; BL: baseline; CNV = choroidal neovascularization; AL = axial length; LE = left eye; logMAR = logarithm of the minimum angle of resolution; LP = laser photocoagulation; PDT = photodynamic therapy; RE = right eye; SD = standard deviation.

**Table 2:** Genotype distribution, minor allele frequency, and Hardy–Weinberg equilibrium analysis for each studied SNP in our population.

SNP	TOTAL *n* = 215					Control Group *n* = 116					Study Group *n* = 99					Control vs. Study
	Genotypes *n* (%)			MAF	H-W	Genotypes *n* (%)			MAF	H-W	Genotypes *n* (%)			MAF	H-W	*p*-Value
	Wt	Het	Hom			Wt	Het	Hom			Wt	Het	Hom			
*KDR* G > A	114	90	11	0.261	0.29	59	51	6	0.272	0.35	55	39	5	0.247	0.79	0.783
rs2239702	(53.02)	(41.86)	(5.12)			(50.9)	(44.0)	(5.2)			(55.6)	(39.4)	(5.1)			
*KDR* C > T	166	48	1	0.116	0.32	92	24	0	0.103	0.61	74	24	1	0.131	1	0.445
rs2305948	(77.21)	(22.33)	(0.47)			(79.3)	(20.7)	(0.0)			(74.7)	(24.2)	(1.0)			
*KDR* C > T	52	115	48	0.491	0.34	26	66	24	0.491	0.19	26	49	24	0.490	1	0.555
rs7667298	(24.19)	(53.49)	(22.33)			(22.4)	(56.9)	(20.7)			(26.3)	(49.5)	(24.2)			
*KDR* T > A	125	79	11	0.235	0.85	63	45	8	0.263	1	62	34	3	0.202	0.76	0.289
rs1870377	(58.14)	(36.74)	(5.12)			(54.3)	(38.8)	(6.9)			(62.6)	(34.3)	(3.0)			
*KDR* C > T	51	116	48	0.493	0.28	25	68	23	0.491	0.10	26	48	25	0.495	0.84	0.329
rs2071559	(23.72)	(53.95)	(22.33)			(21.6)	(58.6)	(19.8)			(26.3)	(48.5)	(25.3)			
*FLT1* C > T	183 (85.12)	31	1	0.077	1	96	19	1	0.091	1	87	12	0	0.061	1	0.430
rs664393		(14.42)	(0.47)			(82.8)	(16.4)	(0.9)			(87.9)	(12.1)	(0.0)			
*FLT1* A > G	128	80	7	0.219	0.23	68	43	5	0.228	0.79	60	37	2	0.207	0.23	0.639
rs7993418	(59.53)	(37.21)	(3.26)			(58.6)	(37.1)	(4.3)			(60.6)	(37.4)	(2.0)			
*FLT1* C > A	73	103	39	0.421	0.78	33	65	18	0.435	0.19	40	38	21	0.404	0.06	0.035
rs9554320	(33.95)	(47.91)	(18.14)			(28.4)	(56.0)	(15.5)			(40.4)	(38.4)	(21.2)			
*FLT1* A > C	100	98	17	0.307	0.34	51	56	9	0.319	0.29	49	42	8	0.293	1	0.684
rs9582036	(46.51)	(45.58)	(7.91)			(44.0)	(48.3)	(7.8)			(49.5)	(42.4)	(8.1)			

SNP = single nucleotide polymorphism; Wt = wildtype genotype; Het = heterozygous genotype; Hom = homozygous genotype; MAF = minor allele frequency; H-W = *p*-value for the Hardy–Weinberg equilibrium analysis.

**Table 3:** Association study of *FLT1* genotypes with BCVA improvement/worsening at 12 months.

**SNP**	**Genotype**	**Improvement**						
		**YES** **n (%)**	**NO** **n (%)**	**Genetic Model** **(Reference)**	**OR (95%CI)**	***p*** **-Value**	**AIC**	**BIC**
*FLT1* A > G rs7993418	A/A	50 (61.7)	18 (58.1)	Codominant (AA) a	1.11 (0.47–2.62)	0.770	137.6	145.8
	G/A	30 (37)	12 (38.7)	Codominant (AA) b	2.78 (0.16–46.78)			
	G/G	1 (1.2)	1 (3.2)	Dominant (AA)	1.16 (0.50–2.70)	0.720	136	141.4
				Recessive (GG)	0.38 (0.02–6.19)	0.500	135.7	141.1
				Log-additive	0.82 (0.38–1.78)	0.610	135.9	141.3
*FLT1* C > A rs9554320	C/C	33 (40.7)	11 (35.5)	Codominant (CC) c	1.16 (0.45–3.01)	0.820	137.7	145.9
	C/A	31 (38.3)	12 (38.7)	Codominant (CC) d	1.41 (0.48–4.17)			
	A/A	17 (21)	8 (25.8)	Dominant (CC)	1.25 (0.53–2.95)	0.610	135.9	141.3
				Recessive (AA)	0.76 (0.29–2.01)	0.590	135.8	141.3
				Log-additive	0.84 (0.49–1.44)	0.530	135.7	141.2
*FLT1* A > C rs9582036	A/A	41 (50.6)	15 (48.4)	Codominant (AA) e	0.81 (0.33–1.99)	0.091	133.3	141.5
	C/A	37 (45.7)	11 (35.5)	Codominant (AA) f	4.56 (0.97–21.44)			
	C/C	3 (3.7)	5 (16.1)	Dominant (AA)	1.09 (0.48–2.50)	0.830	136.1	141.5
				Recessive (CC)	0.20 (0.04–0.90)	0.032	131.6	137
				Log-additive	0.69 (0.36–1.33)	0.270	134.9	140.4
**SNP**	**Genotype**	**Worsening**						
		**YES** **n (%)**	**NO** **n (%)**	**Genetic model**	**OR (95%CI)**	***p*** **-value**	**AIC**	**BIC**
*FLT1* A > G rs7993418	A/A	6 (100)	62 (58.5)	Codominant (AA) g	NA (0.00-NA)	0.045	46.6	54.7
	G/A	0 (0)	42 (39.6)	Codominant (AA) h	NA (0.00-NA)			
	G/G	0 (0)	2 (1.9)	Dominant (AA)	NA (0.00-NA)	0.013	44.6	50
				Recessive (GG)	0.00 (0.00-NA)	0.640	50.6	56
				Log-additive	0.00 (0.00-NA)	0.013	44.6	50
*FLT1* C > A rs9554320	C/C	3 (50)	41 (38.7)	Codominant (CC) i	0.98 (0.19–5.12)	0.210	49.7	57.8
	C/A	3 (50)	40 (37.7)	Codominant (CC) j	NA (0.00-NA)			
	A/A	0 (0)	25 (23.6)	Dominant (CC)	1.59 (0.31–8.23)	0.580	50.5	55.9
				Recessive (AA)	0.00 (0.00-NA)	0.077	47.7	53.1
				Log-additive	0.51 (0.15–1.77)	0.260	49.5	55
*FLT1* A > C rs9582036	A/A	4 (66.7)	52 (49.1)	Codominant (AA) k	1.77 (0.31–10.11)	0.510	51.4	59.6
	C/A	2 (33.3)	46 (43.4)	Codominant (AA) l	NA (0.00-NA)			
	C/C	0 (0)	8 (7.5)	Dominant (AA)	2.08 (0.36–11.83)	0.400	50.1	55.5
				Recessive (CC)	0.00 (0.00-NA)	0.340	49.9	55.3
				Log-additive	0.47 (0.10–2.27)	0.310	49.8	55.2

SNP: Single Nucleotide Polymorphism; OR: Odds Ratio; CI: Confidence Interval; AIC: Akaike information criterion; BIC: Bayesian information criterion; NA: Not applicable; a: A/A vs. G/A; b: A/A vs. G/G; c: C/C vs. C/A; d: C/C vs. A/A; e: A/A vs. A/C; f: A/A vs. C/C; g: A/A vs. G/A; h: A/A vs. G/G; i: C/C vs. C/A; j: C/C vs. A/A; k: A/A vs. C/A; l: A/A vs. C/C; bold: *p*-value < 0.05 of genetic models explaining the association between SNP and response.

**Table 4:** Association study of *KDR* genotypes with BCVA improvement/worsening at 12 months.

SNP	Genotype	Improvement						
		YES n (%)	NO n (%)	Genetic Model (Reference)	OR (95%CI)	*p*-Value	AIC	BIC
*KDR* G > A rs2239702	G/G	44 (54.3)	18 (58.1)	Codominant (GG) a	0.89 (0.38–2.10)	0.890	137.9	146.1
	G/A	33 (40.7)	12 (38.7)	Codominant (GG) b	0.61 (0.06–5.85)			
	A/A	4 (4.9)	1 (3.2)	Dominant (GG)	0.86 (0.37–1.98)	0.720	136	141.4
				Recessive (AA)	1.56 (0.17–14.52)	0.690	136	141.4
				Log-additive	1.18 (0.57–2.43)	0.660	135.9	141.4
*KDR* C > T rs2305948	C/C	57 (70.4)	27 (87.1)	Codominant (CC) c	0.37 (0.12–1.17)	0.140	134.1	142.3
	T/C	23 (28.4)	4 (12.9)	Codominant (CC) d	0.00 (0.00-NA)			
	T/T	1 (1.2)	0 (0)	Dominant (CC)	0.35 (0.11–1.11)	0.055	132.5	137.9
				Recessive (TT)	NA (0.00-NA)	0.420	135.5	140.9
				Log-additive	2.82 (0.91–8.76)	**0.049**	132.3	137.7
*KDR* C > T rs7667298	C/C	19 (23.5)	10 (32.3)	Codominant (CC) e	0.48 (0.17–1.31)	0.200	134.9	143.1
	T/C	44 (54.3)	11 (35.5)	Codominant (CC) f	1.06 (0.36–3.13)			
	T/T	18 (22.2)	10 (32.3)	Dominant (CC)	0.64 (0.26–1.60)	0.350	135.3	140.7
				Recessive (TT)	0.60 (0.24–1.50)	0.280	135	140.4
				Log-additive	0.98 (0.55–1.74)	0.930	136.1	141.6
*KDR* T > A rs1870377	T/T	48 (59.3)	23 (74.2)	Codominant (TT) g	0.58 (0.23–1.46)	0.130	134.1	142.2
	T/A	29 (35.8)	8 (25.8)	Codominant (TT) h	0.00 (0.00-NA)			
	A/A	4 (4.9)	0 (0)	Dominant (TT)	0.51 (0.20–1.27)	0.140	133.9	139.3
				Recessive (AA)	NA (0.00-NA)	0.100	133.5	138.9
				Log-additive	2.05 (0.88–4.79)	0.080	133.1	138.5
*KDR* C > T rs2071559	C/C	19 (23.5)	12 (38.7)	Codominant (CC) i	0.32 (0.12–0.90)	0.051	132.2	140.3
	T/C	44 (54.3)	9 (29)	Codominant (CC) j	0.88 (0.31–2.53)			
	T/T	18 (22.2)	10 (32.3)	Dominant (CC)	0.49 (0.20–1.18)	0.110	133.6	139.1
				Recessive (TT)	0.60 (0.24–1.50)	0.280	135	140.4
				Log-additive	1.10 (0.62–1.96)	0.730	136	141.5
**Worsening**								
*KDR* G > A rs2239702	G/G	1 (16.7)	61 (57.5)	Codominant (GG) k	0.17 (0.02–1.56)	0.100	48.2	56.4
	G/A	4 (66.7)	41 (38.7)	Codominant (GG) l	0.07 (0.00–1.25)			
	A/A	1 (16.7)	4 (3.8)	Dominant (GG)	0.15 (0.02–1.31)	**0.044**	46.7	52.2
				Recessive (AA)	5.10 (0.48–54.45)	0.240	49.4	54.8
				Log-additive	4.03 (1.06–15.28)	**0.037**	46.5	51.9
*KDR* C > T rs2305948	C/C	5 (83.3)	79 (74.5)	Codominant (CC) m	1.65 (0.18–14.74)	0.850	52.5	60.6
	T/C	1 (16.7)	26 (24.5)	Codominant (CC) n	NA (0.00-NA)			
	T/T	0 (0)	1 (0.9)	Dominant (CC)	1.71 (0.19–15.29)	0.610	50.5	56
				Recessive (TT)	0.00 (0.00-NA)	0.740	50.7	56.1
				Log-additive	0.58 (0.07–4.90)	0.590	50.5	55.9
*KDR* C > T rs7667298	C/C	0 (0)	29 (27.4)	Codominant (CC) o	0.00 (0.00-NA)	0.160	49.1	57.2
	T/C	4 (66.7)	51 (48.1)	Codominant (CC) p	0.00 (0.00-NA)			
	T/T	2 (33.3)	26 (24.5)	Dominant (CC)	0.00 (0.00-NA)	0.054	47.1	52.5
				Recessive (TT)	1.54 (0.27–8.89)	0.640	50.6	56
				Log-additive	2.10 (0.61–7.19)	0.220	49.3	54.7
*KDR* T > A rs1870377	T/T	4 (66.7)	67 (63.2)	Codominant (TT) q	1.04 (0.18–5.99)	0.800	52.3	60.5
	T/A	2 (33.3)	35 (33)	Codominant (TT) r	NA (0.00-NA)			
	A/A	0 (0)	4 (3.8)	Dominant (TT)	1.16 (0.20–6.65)	0.860	50.8	56.2
				Recessive (AA)	0.00 (0.00-NA)	0.500	50.3	55.8
				Log-additive	0.78 (0.16–3.77)	0.750	50.7	56.1
*KDR* C > T rs2071559	C/C	2 (33.3)	29 (27.4)	Codominant (CC) s	0.84 (0.15–4.90)	0.170	49.2	57.3
	T/C	4 (66.7)	49 (46.2)	Codominant (CC) t	NA (0.00-NA)			
	T/T	0 (0)	28 (26.4)	Dominant (CC)	1.33 (0.23–7.64)	0.750	50.7	56.1
				Recessive (TT)	0.00 (0.00-NA)	0.059	47.2	52.7
				Log-additive	0.52 (0.16–1.76)	0.280	49.6	55.1

SNP = single nucleotide polymorphism; OR = odds Ratio; CI = confidence Interval; AIC = Akaike information criterion; BIC = Bayesian information criterion; NA = not applicable; a: G/G vs. G/A; b: G/G vs. A/A; c: C/C vs. T/C; d: C/C vs. T/T; e: C/C vs. T/C; f: C/C vs. T/T; g: T/T vs. T/A; h: T/T vs. A/A; i: C/C vs. T/C; j: C/C vs. T/T; k: G/G vs. G/A; l: G/G vs. A/A; m: C/C vs. T/C; n: C/C vs. T/T; o: C/C vs. T/C; p: C/C vs. T/T; q: T/T vs. T/A; r: T/T vs. A/A; s: C/C vs. T/C; t: C/C vs. T/T; bold: *p*-value < 0.05 of genetic models explaining the association between SNP and response.

**Table 5:** Alleles association study with BCVA improvement/worsening at 12 months.

SNP Major > Minor	Allele	Improvement				Worsening			
		YES n (%)	NO n (%)	OR (95%CI)	*p*-Value	YES n (%)	NO n (%)	OR (95%CI)	*p*-Value
*FLT1* rs664393 C > T	T	10 (6.2)	4 (6.5)	0.95 (0.29–3.16)	1 Fischer	1 (8.3)	13 (6.1)	1.39 (0.17–11.62)	0.548 Fischer
	C	152 (93.8)	58 (93.5)			11 (91.7)	199 (93.9)		
*FLT1* rs7993418 A > G	G	32 (19.8)	14 (22.6)	0.84 (0.41–1.72)	0.639	0 (0)	46 (21.7)	0 (0.0-NA)	0.133 Fischer
	A	130 (80.2)	48 (77.4)			12 (100)	166 (78.3)		
*FLT1* rs9554320 C > A	A	65 (40.1)	28 (45.2)	0.81 (0.45–1.47)	0.494	3 (25.0)	90 (42.5)	0.45 (0.12–1.72)	0.367 Fischer
	C	97 (59.9)	34 (54.8)			9 (75.0)	122 (57.5)		
*FLT1* rs9582036 A > C	C	43 (26.5)	21 (33.9)	0.71 (0.38–1.33)	0.277	2 (16.7)	62 (29.2)	0.48 (0.1–2.27)	0.516 Fischer
	A	119 (73.5)	41 (66.1)			10 (83.3)	150 (70.8)		
*KDR* rs2239702 G > A	A	41 (25.3)	14 (22.6)	1.16 (0.58–2.32)	0.671	6 (50.0)	49 (23.1)	3.33 (1.03–10.78)	**0.035**
	G	121 (74.7)	48 (77.4)			6 (50.0)	163 (76.9)		
*KDR rs2305948* C > T	T	25 (15.4)	4 (6.5)	2.65 (0.88–7.94)	0.073	1 (8.3)	28 (13.2)	0.6 (0.07–4.81)	1 Fischer
	C	137 (84.6)	58 (93.5)			11 (91.7)	184 (86.8)		
*KDR* rs7667298 T > C	C	82 (50.6)	31 (50.0)	1.03 (0.57–1.84)	0.934	4 (33.3)	109 (51.4)	0.47 (0.14–1.62)	0.251 Fischer
	T	80 (49.4)	31 (50.0)			8 (66.7)	103 (48.6)		
*KDR* rs1870377 T > A	A	37 (22.8)	8 (12.9)	2 (0.87–4.57)	0.097	2 (16.7)	43 (20.3)	0.79 (0.17–3.72)	1 Fischer
	T	125 (77.2)	54 (87.1)			10 (83.3)	169 (79.7)		
*KDR* rs2071559 *C > T*	C	82 (50.6)	33 (53.2)	0.9 (0.5–1.62)	0.727	8 (66.7)	107 (50.5)	1.96 (0.57–6.71)	0.376 Fischer
	T	80 (49.4)	29 (46.8)			4 (33.3)	105 (49.5)		

OR = odds ratio; CI = confidence interval; NA = not applicable; bold = *p* < 0.05.

## Data Availability

The data presented in this study are available on request from the corresponding author. The data are not publicly available due to containing clinical and personal information.

## Supplementary Materials

The following supporting information can be downloaded at: https://www.mdpi.com/article/10.3390/pharmaceutics14081555/s1, Figure S1: *FLT1* linkage disequilibrium analysis; Figure S2: *KDR* linkage disequilibrium analysis; Table S1: Haplotype frequencies of *FLT1* variants and association with response; Table S2: Haplotype frequencies of *KDR* variants and association with response.
