# Supplementary material for: Genetic Polymorphisms in VEGFR Coding Genes (FLT1/KDR) on Ranibizumab Response in High Myopia and Choroidal Neovascularization Patients
Source: Pharmaceutics. 2022 Jul 26;14(8):1555. doi: 10.3390/pharmaceutics14081555 (PMC9330346; doi:10.3390/pharmaceutics14081555)
Supplement: Supplementary file 1 [file pharmaceutics-14-01555-s001.zip › pharmaceutics-1802538-supplementary.pdf]

Supplementary Materials: Genetic Polymorphisms in VEGFR Coding Genes (*FLT1*/*KDR*) on Ranibizumab Response in High Myopia and Choroidal Neovascularization Patients

David Blánquez-Martínez, Xando Díaz-Villamarín, Sonia García-Rodríguez, Alba Antúnez-Rodríguez, Ana Pozo-Agundo, Luis Javier Martínez-González, José Ignacio Muñoz-Ávila and Cristina Lucía Dávila -Fajardo

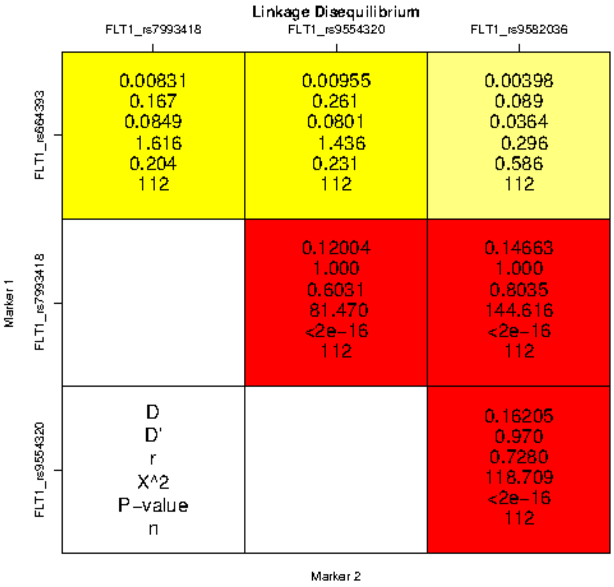

Figure S1. Linkage disequilibrium (LD) analysis of FLT1 genetic variants.

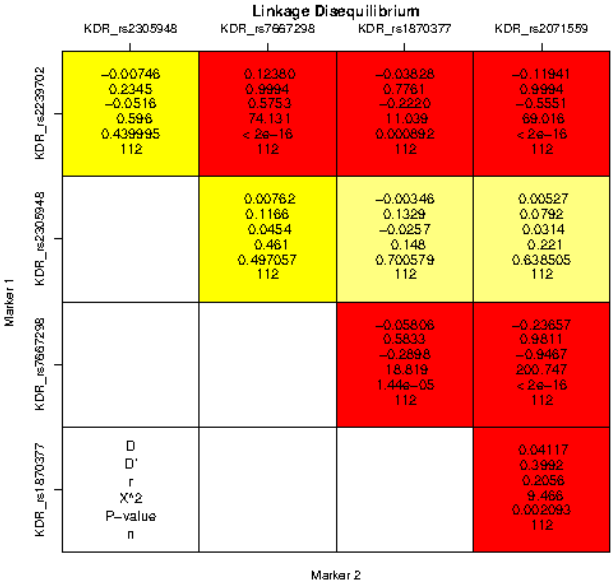

Figure S2. Linkage disequilibrium (LD) analysis of KDR genetic variants.

Table S1. Haplotypes frequencies of FLT1 variants and association with response.

* Id	rs664393	rs7993418	rs9554320	rs9582036	Total	BCVA Improvement		OR (95% CI)	*p*-value
						YES	NO		
1	C	A	C	A	0.555	0.575	0.486	reference	-
2	C	G	A	C	0.182	0.172	0.226	1.65 (0.72 – 3.80)	0.24
3	C	A	A	A	0.121	0.127	0.111	0.85 (0.32 – 2.24)	0.75
4	C	A	A	C	0.076	0.057	0.113	3.05 (0.95 – 9.78)	0.064
*** Id**	**rs664393**	**rs7993418**	**rs9554320**	**rs9582036**	**Total**	**BCVA Worsening**		**OR (95% CI)**	***p*-value**
						**YES**	**NO**		
1	C	A	C	A	0.555	0.667	0.550	reference	-
2	C	G	A	C	0.182	NA	0.193	NA	
3	C	A	A	A	0.121	0.083	0.121	NA	
4	C	A	A	C	0.076	0.167	0.070	NA	

* Only shows haplotypes with frequency higher than 0.1 in at least one group; rs: Reference single nucleotide polymorphism (SNP); BCVA: Best-corrected visual acuity; OR: Odds ratio.

Table S2. Haplotypes frequencies of KDR variants and association with response.

Id	rs2239702	rs2305948	rs7667298	rs1870377	rs2071559	Total	BCVA Improvement		OR (95% CI)	*p*-value
							YES	NO		
1	G	C	C	T	T	0.295	0.293	0.323	reference	-
2	A	C	T	T	C	0.219	0.215	0.226	0.86 (0.35 – 2.08)	0.730
3	G	C	T	T	C	0.173	0.134	0.258	2.15 (0.90 – 5.15)	0.089
4	G	C	C	A	T	0.119	0.133	0.097	0.68 (0.21 – 2.20)	0.520
*** Id**	**rs2239702**	**rs2305948**	**rs7667298**	**rs1870377**	**rs2071559**	**Total**	**BCVA Worsening**		**OR (95% CI)**	***p*-value**
							**YES**	**NO**		
1	G	C	C	T	T	0.295	0.083	0.305	reference	-
2	A	C	T	T	C	0.219	0.500	0.205	0.09 (0.01 – 0.86)	0.039
3	G	C	T	T	C	0.173	0.167	0.174	0.41 (0.04 – 4.39)	0.46
4	G	C	C	A	T	0.119	0.167	0.117	0.17 (0.01 – 2.13)	0.17

* Only shows haplotypes with frequency higher than 0.1 in at least one group; rs: Reference single nucleotide polymorphism (SNP); BCVA: Best-corrected visual acuity; OR: Odds ratio.
